# Near‐Infrared Chemiluminescent Carbon Nanodots and Their Application in Reactive Oxygen Species Bioimaging

**DOI:** 10.1002/advs.201903525

**Published:** 2020-03-09

**Authors:** Cheng‐Long Shen, Qing Lou, Jin‐Hao Zang, Kai‐Kai Liu, Song‐Nan Qu, Lin Dong, Chong‐Xin Shan

**Affiliations:** ^1^ Henan Key Laboratory of Diamond Optoelectronic Materials and Devices Key Laboratory of Materials Physics Ministry of Education School of Physics and Microelectronics Zhengzhou University Zhengzhou 450052 China; ^2^ Joint Key Laboratory of the Ministry of Education Institute of Applied Physics and Materials Engineering University of Macau Macau 999078 China

**Keywords:** bioimaging, carbon nanodots, chemiluminescence, reactive oxygen species, sensors, turn‐on probes

## Abstract

Reactive oxygen species (ROS) are generated in the body and related to many pathophysiological processes. Hence, detection of ROS is indispensable in understanding, diagnosis, and treatment of many diseases. Here, near‐infrared (NIR) chemiluminescent (CL) carbon nanodots (CDs) are fabricated for the first time and their CL quantum yield can reach 9.98 × 10^−3^ einstein mol^−1^, which is the highest value ever reported for CDs until now. Nanointegration of NIR CDs and peroxalate (P‐CDs) through the bridging effect of amphiphilic triblock copolymer can serve as turn‐on probes for the detection and imaging of hydrogen peroxide (H_2_O_2_). Considering high efficiency and large penetration depth of NIR photons, the P‐CDs are employed in bioimaging H_2_O_2_ in vitro and in vivo, and the detection limit can reach 5 × 10^−9^ m, among the best reported of CDs‐based sensors. Moreover, imaging of inflammatory H_2_O_2_ in a mouse model of peritonitis is achieved by employing the P‐CDs as sensors. The results may provide a clue for the diagnosis and treatment of inflammation or cancers employing CL CDs as sensors.

## Graphical abstract

NIR carbon nanodots (CDs) with efficient chemiluminescence are developed. Nanointegration of NIR CDs and peroxalate can serve as turn‐on probes for bioimaging of exogenous and endogenous H_2_O_2_ in vitro and in vivo with the detection limit is 5 × 10^−9^ m. Bioimaging inflammatory H_2_O_2_ in a mouse model of peritonitis is achieved.

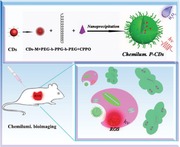

## 1 Introduction

Reactive oxygen species (ROS), as significant reactive and signaling molecules in the process of intravital metabolism, play a great role in many biological processes.^[^ ^1^, ^2^, ^3^, ^4^ ^]^ Among the ROS molecules, hydrogen peroxide (H_2_O_2_) is prime reactive species, whose overproduction is closely related to various diseases, including inflammation, cancer, or neurological diseases.^[^ ^5^, ^6^, ^7^, ^8^, ^9^ ^]^ Thus it is of great importance in monitoring the concentration of H_2_O_2_ in living specimens. To this end, a number of mapping tools including “dark” biological processes and radiative recombination mechanism have been developed. Chemiluminescence (CL), a kind of light emission induced by energy transfer from chemical reactions, has evoked considerable interest as one ultrasensitive chemical analysis method with quantification and localization.^[^ ^10^, ^11^, ^12^ ^]^ Without auto‐fluorescent interference and phototoxicity from high‐energy excitation light, CL shows high signal‐to‐noise ratio and low perturbation in sensing H_2_O_2_ in vivo compared to photoluminescence (PL) method.^[^ ^13^, ^14^, ^15^ ^]^ Moreover, unlike the bioluminescence probe requiring the activation of bioactive enzyme, CL imaging of H_2_O_2_ is a process of nonenzymatic reaction employing a H_2_O_2_‐responsive peroxalate that can transfer chemical energy to the CL emitter. Nevertheless, current CL reporters on H_2_O_2_ concentrate mainly on small‐molecular dyes, semiconducting polymer, and aggregation induced emission nanoparticles, which suffer from low efficiency, short emission wavelength, and low chemical stability in highly oxidative ROS.^[^ ^16^, ^17^ ^]^ Thereby, it is meaningful to develop new class of CL nanosensors for the imaging and detecting ROS in vitro and in vivo.

Carbon nanodots (CDs), which are considered as discrete quasi‐spherical nanoparticles with sizes less than 10 nm, are one kind of promising nanomaterials in bioimaging, photocatalysis, optoelectronics, and sensing owing to its unique properties such as high emission efficiency, good biocompatibility, high photo‐stability, and tunable luminescence.^[^ ^18^, ^19^, ^20^, ^21^, ^22^, ^23^, ^24^, ^25^, ^26^, ^27^, ^28^ ^]^ Recently, the CL properties of CDs in peroxlate–H_2_O_2_ system have been investigated, and it has been found that multicolor bright and persistent CL can be obtained from CDs.^[^ ^29^ ^]^ Therefore, it is practicable to develop versatile CL probes based on CDs to detect ROS via in vivo or in vitro imaging. In general, there are several advantages for CDs as the CL imaging probes: (i) CDs exhibit excellent light emission ability and the luminescence property can be tuned by different methods;^[^ ^30^, ^31^ ^]^ (ii) CDs with emission wavelength at near‐infrared (NIR) region (beyond 650 nm) can be synthesized, which is particularly important for bioimaging due to the deep tissue penetration;^[^ ^32^, ^33^ ^]^ (iii) the energy level of CDs can be modulated to reduce the energy interval with the energetic intermediate‐1,2‐dioxetanedione, which is beneficial to the electron transfer in the process of H_2_O_2_‐activated luminescence and further enhances the CL quantum efficiency (QY) of CDs;^[^ ^14^ ^]^ (iv) the abundant surface radicals endow CDs with the good ability of drug carriers;^[^ ^34^, ^35^ ^]^ (v) benign biocompatibility and high photostability of CDs make them promising application in CL bioimaging.^[^ ^36^, ^37^, ^38^, ^39^, ^40^, ^41^, ^42^ ^]^ Hence, it is significant to develop CD‐based CL probes to generate luminescent signals in response to H_2_O_2_ and further provides a category of bioimaging for ROS.

Here, CDs with NIR CL have been synthesized with solvothermal method employing citric acid and urea as precursors in *N,N*‐diethylformamide (DEF). The CDs exhibit an QY of 9.98 × 10^−3^ einstein mol^−1^ in bis(2,4,5‐trichloro‐6‐carbopentoxyphenyl) oxalate (CPPO) and H_2_O_2_ solution, which is the highest value ever reported for the CL from CDs. In addition, P‐CDs are transformed from NIR CDs and CPPO through the nanoscopic coaggregation process in the existence of amphiphilic polymeric conjugate (PEG‐b‐PPG‐b‐PEG). By virtue of the high efficiency and deep penetration of NIR emission, the P‐CDs have been employed to bioimaging H_2_O_2_ in vitro and in vivo. Moreover, imaging of inflammatory H_2_O_2_ in a mouse model of peritonitis has been achieved by employing the P‐CD as a sensor. The present work exhibits considerable potential for CDs as new class of CL probes for in vivo imaging applications in the diagnosis and treatment of inflammation or cancers.

## 2 Results and Discussion

In our previous report, it is found that multicolor PL and CL can be obtained from CDs by changing the degree of graphitization of the conjugated sp^2^‐domains, implying that it is promising to excavate the application of CDs as novel probes for ROS sensing.^[^ ^29^ ^]^ However, a high CL QY and NIR emission of CD based probes are still urgent to be developed.

**Figure 1:**
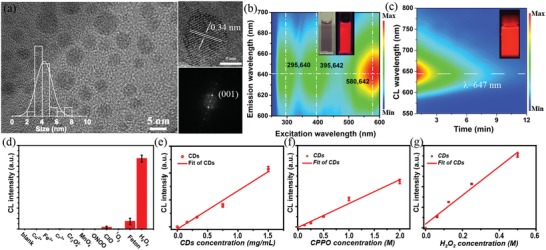
a) Transmission electron microscopy (TEM) images, high‐resolution TEM (HRTEM) images, and selected area electron diffraction (SAED) pattern of the carbon nanodots (CDs) (inset: the size distribution of the CDs). b) 3D fluorescence mapping of the CDs in aqueous solution. (inset: the fluorescence image of the CDs under sunlight (left) and 365 nm UV excitation (right)). c) The decay spectra of the chemiluminescent (CL) from the CDs (inset: the photograph of the CL from the CDs). d) CL response of the CDs to various reactive oxygen species (ROS) in CL analysis instrument. CL signal intensities response of CDs to the concentration of e) CD, f) CPPO, and g) H_2_O_2_.

In this report, NIR CDs have been prepared with citric (1 g) and urea (2 g) in 10 mL DEF by one‐step solvothermal strategy (160 °C for 8 h). DEF, as polar aprotic solvents, can exacerbate dehydration reaction and increase the conjugation degree of CDs due to effective dehydration reaction happened between citric acid and intramolecules with longer carbon chains (ethyl group from DEF).^[^ ^29^, ^40^ ^–42]^ Transmission electron microscopy (TEM) has been used to characterize the morphology of the CDs, as shown in **Figure** **1**a, where the CDs display a broad particle size distribution with an average diameter of around 4 nm. The high‐resolution TEM (HRTEM) image and selected area electron diffraction (SAED) pattern reveal that the CDs have well‐resolved lattice spacing of 0.34 nm, which corresponds to the (002) crystallographic plane of graphitic carbon. The X‐ray diffraction (XRD) patterns of the CDs show a main peak at 23.1°, which can be attributed to the (002) planes of graphitic carbon (Figure S1, Supporting Information). The TEM and XRD results reflect the good crystalline structure of the CDs. Raman spectra also verify a high degree of crystallinity in the CDs owing to a large ratio of G band (1580 cm^−1^) and D band (1350 cm^−1^) of about 0.99 (Figure S2, Supporting Information). In addition, the deconvoluted high‐resolution X‐ray photoelectron spectra (XPS) for C1s, N1s, and O1s reveal that there are O‐ and N‐containing functional groups on the surface of the CDs, which facilitates the surface modification for the CDs (Figures S3−S6, Supporting Information). The PL and CL properties of the CDs are illustrated in Figure 1b,c. Under the excitation of a 365 nm UV lamp, the aqueous solution of the CDs exhibits bright deep‐red luminescence with the maximum PL emission peak at around 642 nm, extended to the NIR region (>650 nm). The excitation−emission 3D mapping pattern of the CDs shown in Figure 1b indicates that the emission center is almost immobile and ranged with three maximum excitation peaks at 295, 395, and 580 nm, which can be assigned to π−π* transition of sp^2^ C, n−π* transition of conjugated C**=**O and Mie scattering due to the extended conjugation in the CD structure, respectively. The PL lifetime of 5.9 ns for the CDs can be calculated and obtained with a single exponential function (Figure S7, Supporting Information). Moreover, the CL emission spectra (≈647 nm) after adding CDs into CPPO and H_2_O_2_ solution present consistent spectra with the PL spectra, implying that the CD‐based CL is originated from direct radiative recombination of the CDs under the chemical excitation (Figure 1c and Figures S8, S9, and Table S1, Supporting Information). The CL QY of the CDs is calculated to be 9.98 × 10^−3^ einstein mol^−1^ using lucigenin as the reference, which is the highest value ever reported even in CDs‐based CL systems (Figures S10−S12 and Table S2, Supporting Information; **Table** **1**). Meanwhile, the CL QY of the CDs in this work is much higher than the small‐molecule dye, metal nanoparticles and is comparable to the semiconductor polymer nanoparticles (SPNs). The selectivity of the CL from CDs for the detection of H_2_O_2_ is further studied with CL analysis instrument. In the presence of H_2_O_2_, the CL signals of the CDs are far higher (>50 times) than that in the presence of other ROS, verifying that the CL system based on the CDs has a high selectivity to H_2_O_2_ (Figure 1d). Additionally, the CL intensity of the CDs is directly proportional to the concentration of the CDs, CPPO, and H_2_O_2_, exhibiting the feasibility of quantification analysis of CL for probing H_2_O_2_ (Figure 1e−h and Figures S13−S16, Supporting Information). The efficient NIR CL and high selectivity promise the potential applications of the CDs as a CL probe to H_2_O_2_.

**Table 1:** Photoluminescence (PL) and chemiluminescent (CL) characteristics of the carbon nanodots (CDs)

Samples	λ_em_ ^a)^ [nm]	τ^b)^ [ns]	λ_em_ ^c)^ [nm]	Φ_C_ ^d)^ [einstein mol^−1^]
CDs	642	5.9	647	9.98 × 10^−3^

a) PL maximum peak

b) PL lifetime

c) CL maximum peak

d) CL quantum yield.

**Figure 2:**
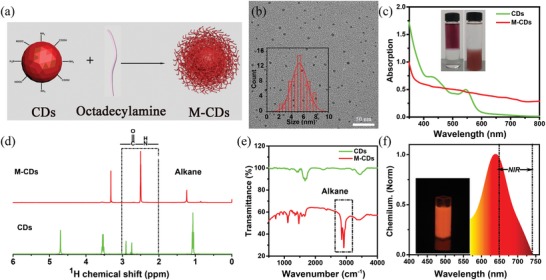
a) Schematic illustration of the preparation of the M‐CDs. b) Transmission electron microscopy (TEM) images of the M‐CDs (inset: the size distribution of the M‐CDs). c) The UV–Vis absorption spectra of the CDs in aqueous and M‐CDs in CH_2_Cl_2_ (inset: the distribution of CDs and M‐CDs in H_2_O−CH_2_Cl_2_). d) ^1^H NMR spectra of the CDs in D_2_O and M‐CDs in CDCl_3_. e) Fourier transform infrared spectroscopy (FTIR) spectra of the CDs and M‐CDs. f) Emission spectra after adding M‐CDs solution into CPPO and H_2_O_2_ solution (inset: the image of the chemiluminescent (CL) M‐CDs).

For a better coaggregating CPPO (peroxalate esters) and hydrophilic CDs to form nanointegrated CDs (P‐CDs), we have first developed one facile method to modulate CDs with lipophilic long chain (Figure S17, Supporting Information). As shown in **Figure** **2**a, the water‐soluble CDs can be modified by octadecylamine to synthesize oil‐soluble NIR CDs (M‐CDs) for using nanoscopic coaggregation. In the process, the M‐CDs were formulated with CDs, octadecylamine, and mPEG‐NH_2_ in dimethylformamide (DMF) as illustrated in Experimental Section. With dichloromethane (CH_2_Cl_2_) as eluent, the hydrophobic M‐CDs can be extracted from DMF with massive water and kept in dichloromethane (CH_2_Cl_2_) solution. As illustrated in the TEM images, the as‐prepared M‐CDs are spherical nanoparticles with an average diameter of around 5 nm, which is similar to the CDs shown in Figure 2b. As shown in Figure 2c, the UV‐Vis absorption spectra of M‐CDs are red‐shifted compared to initial hydrophilic CDs. This bathochromic shift can be ascribed to the change of functional groups on the surface of the CDs. The inset of Figure 2c illustrates the good phase transfer from the water‐soluble CDs to the oil‐soluble CDs. ^1^H nuclear magnetic resonance (NMR) and Fourier transform infrared spectroscopy (FTIR) have been employed to characterize the modification for the surface functional groups of the CDs and M‐CDs (Figure 2d,e). The intense signals in the range of 2.2–2.9 ppm are corresponding to the protons of amine groups, verifying the substantial amide bond on the surface of M‐CDs. The FTIR peaks at around 2900 cm^−1^ can be ascribed to the stretching vibration of −CH_2_−, which demonstrates the existence of octadecyl chains on the surface of the M‐CDs. The above results indicate that hydrophobic of M‐CDs results from the alkyl‐chain functionalization. In addition, the excitation−emission matrix of the M‐CDs in CH_2_Cl_2_ solution shows identical PL emission and excitation spectral profiles to the initial CDs solution (Figure 1b and Figure S18, Supporting Information). The CL spectra of the M‐CDs also keep consistent with the original CDs, indicating that the modification has no obvious impact on the PL and CL emission properties of the CDs (Figure 2f and Figure S19, Supporting Information).

**Figure 3:**
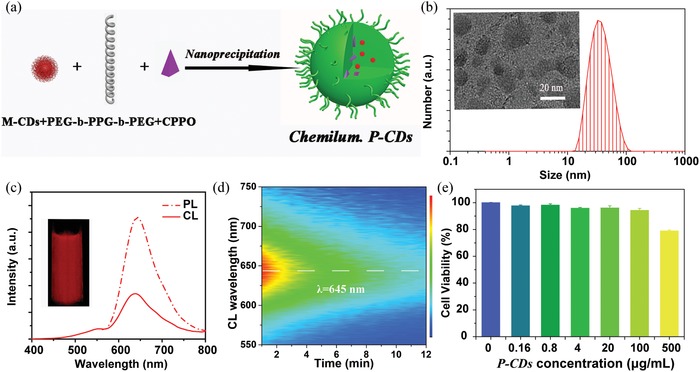
a) Schematic illustration of the preparation of the P‐CDs. b) Dynamic light scattering (DLS) distribution of the P‐CDs (inset: transmission electron microscopy (TEM) images of the P‐CDs). c) The photoluminescence (PL) and CL emission spectra of the P‐CDs, PL spectra is detected under 540 nm excitation for P‐CDs aqueous solution and CL is detected by adding 1 mL, 10 mg mL^−1^ P‐CDs solution into 20 × 10^−3^ m H_2_O_2_ (inset: the photograph of P‐CDs in H_2_O_2_ captured with 30 s). d) The dynamic CL spectra of the P‐CDs. e) Cell viability of Hela cells after 24 h incubation in the different concentration of the P‐CDs.

To explore the CDs as imaging probes for in vitro and in vivo applications, the aqueous dispersion of nanointegrated CDs and peroxalate particles (P‐CDs) have been prepared through the nanoprecipitation of oil‐soluble M‐CDs, CPPO, and amphiphilic PEG‐b‐PPG‐b‐PEG, as illustrated in **Figure** **3**a and Experimental Section. By optimizing the loading weight of CDs in the P‐CDs, the maximum CL QY of the P‐CDs loaded with 2 mg M‐CDs is calculated as about 1.87 × 10^−3^ einstein mol^−1^, which is 7.6 times higher than those loaded with 2 mg water‐soluble CDs (**Table** **2** and Figure S20, Supporting Information). Furthermore, the CL peak intensity of the P‐CDs in water exhibits a 12.8 times enhancement when the surface of the CDs is modified from hydrophilic to hydrophobic, which may originate from the tight bridging between the oil‐soluble M‐CDs and the hydrophobic CPPO through the efficient hydrogen bond interaction. With the further increase in the loading weight of M‐CDs, the P‐CDs exhibit lower CL QYs, which may be generated from the aggregation induced quenching effect due to the strong interaction of high concentrations of M‐CDs (Table 2 and Figures S21 and S22, Supporting Information). As determined by dynamic light scattering (DLS) in Figure 3b and Figures S23 and S24, Supporting Information, the obtained P‐CDs have a number‐weighted hydrodynamic diameter of about 40 nm, which is suitable for the usage in biomedical labeling and diagnostic applications. The TEM and HRTEM images present that P‐CDs have polydispersed morphology with a wide size ranging from 20 to 100 nm, but the average diameter of P‐CDs is about 40 nm (inset of Figure 3b and Figures S25−S27, Supporting Information). This is consistent with the results of DLS. And the well‐resolved lattice spacing of 0.34 nm, attributed to the (002) crystallographic plane of M‐CDs, can also be seen in the P‐CDs. The luminescent property of the P‐CDs in aqueous solution has been characterized by the excitation−emission matrices, illustrating a similar PL property as CDs solution (Figure 3c and Figure S28, Supporting Information). Moreover, the emission spectra after adding P‐CDs into H_2_O_2_ solution have also been measured, as illustrated in Figure 3c, exhibiting a consistent CL emission with the corresponding steady‐state PL spectra. The CL emission of the P‐CDs can persist over tens of minutes (Figure 3d), which is long enough for the in vivo CL imaging experiments. In addition, the biocompatibility of P‐CDs is also investigated, as shown in Figure 3e. More than 80% viability for Hela cells can be obtained when the concentration of the P‐CDs is 500 µg mL^−1^, and indicating a low cytotoxicity, which is beneficial for the CL bioimaging applications.

**Table 2:** Chemiluminescent (CL) QYs of P‐CDs with different M‐CDs in 0.2 m H_2_O_2_

Samples	Φ_C_ ^a)^ [einstein mol^−1^]	Φ_C_ ^a)^ [einsteins mol^−1^]	Φ_C_ ^a)^ [einsteins mol^−1^]	Φ_C_ ^a)^ [einsteins mol^−1^]	Φ_C_ ^a)^ [einsteins mol^−1^]
P‐CDs	0.1 mg	1.0 mg	1.5 mg	2.0 mg	3.0 mg
	2.35 × 10^−5^	2.11 × 10^−4^	2.48 × 10^−4^	1.87 × 10^−3^	1.66 × 10^−4^

a) CL quantum yield.

**Figure 4:**
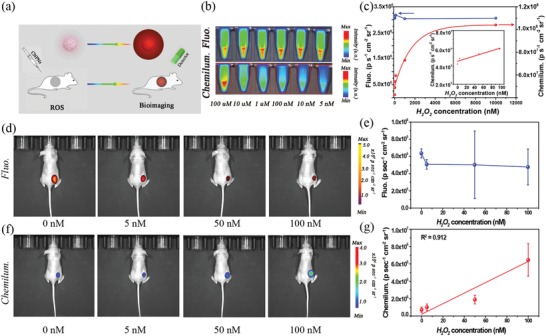
a) Schematic illustration of the exogenous sensing for reactive oxygen species (ROS). b) Photoluminescence (PL) and chemiluminescent (CL) images of P‐CDs in the presence of different concentration of H_2_O_2_. c) PL and CL intensities of P‐CDs under different concentration of H_2_O_2_. d) In vivo PL images of mice with the subcutaneous implantation of different concentration of H_2_O_2_. e) PL intensities as a function of the concentration of H_2_O_2_. f) In vivo CL images of mice with the subcutaneous implantation of different concentration of H_2_O_2_ (*n* = 3 mice per group). g) CL intensities as a function of the concentration of H_2_O_2_ (*n* = 3 mice per group).

With the merit of low toxicity, long persistent glowing, and deep penetration of NIR emission, the P‐CDs can be employed as bioimaging probes to detect H_2_O_2_ in vitro and in vivo. **Figure** **4** **a** illustrates the scheme of in vitro and in vivo CL imaging generated from P‐CDs with the existence of H_2_O_2_ from exogenous environment or endogenous disease. The practicability of P‐CDs for in vitro imaging of exogenous H_2_O_2_ in aqueous has been first assessed by the PL and CL imaging with the IVIS imaging system. The PL and CL images are captured after adding P‐CDs into different concentrations of H_2_O_2_ aqueous solution. As shown in Figure 4b and c, the PL intensity almost remain unchanged even when the addition of H_2_O_2_ is at a high concentration of 5 × 10^−6^ m, implying the PL imaging with P‐CDs is not applicable to the detection of H_2_O_2_. In contrast, the CL intensity of the P‐CDs presents a significant increase with increasing the H_2_O_2_ concentrations, as indicated in Figure 4c, where the CL intensity increases nearly linearly in the range of 0 to 100 × 10^−9^ m with a detection limit of 5 × 10^−9^ m (Figure S29, Supporting Information). The detection limit (5 × 10^−9^ m) is much smaller than the usual concentration of H_2_O_2_ (≈100 × 10^−9^ m) in living bodies, which is the best value ever reported for CDs based probes (Table S3, Supporting Information). To evaluate the ability of in vivo sensing the exogenous H_2_O_2_ for the P‐CDs, the images of PL and CL intensity are also recorded when the anaesthetized nude mice are subcutaneously injected into the dorsal area with the P‐CDs solution and different concentrations of H_2_O_2_. As shown in Figure 4d,e, the PL intensities almost keep constant with the increase of H_2_O_2_ concentration. Nevertheless, the CL intensity increases linearly with the increase of H_2_O_2_ concentration from 0 × 10^−9^ to 100 × 10^−9^ m. Meanwhile, the CL intensity of the P‐CDs with the existence of 5 × 10^−9^ m H_2_O_2_ is about 1.5‐fold higher than that of the P‐CDs alone.

**Figure 5:**
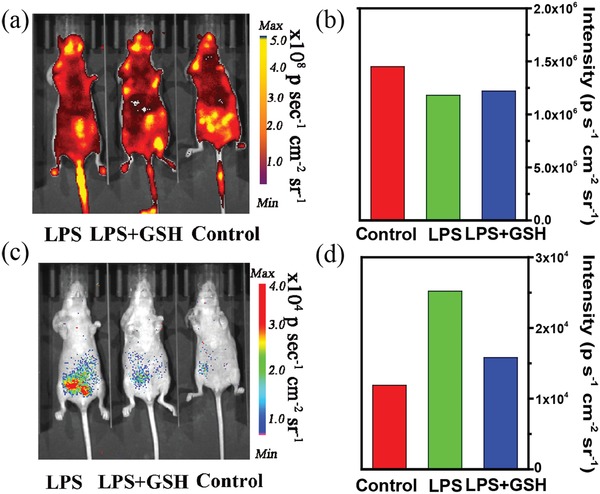
In vivo imaging of endogenous H_2_O_2_ in the mouse model of peritonitis. a) Photoluminescence (PL) and c) chemiluminescent (CL) images of mice intraperitoneally treated with lipopolysaccharide (LPS), LPS plus glutathione (GSH) and saline, followed by an intraperitoneal injection of P‐CDs at *t* = 4 h later. Quantification of b) PL and d) CL intensities for the in vivo images.

Along with the high sensitivity, good biocompatibility, and potential deep tissue penetration, P‐CDs are beneficial for detecting the endogenous H_2_O_2_ due to the abnormal variation in the body of living mice. Endogenous H_2_O_2_ is the significant metabolite when the body suffers from inflammation or cancers. Hence, the early diagnostics and treatment of these diseases can be achieved through monitoring the low concentration endogenous H_2_O_2_. As a proof‐of‐concept, the inflammatory mouse models have been established through intraperitoneal injection of lipopolysaccharide (LPS), which can be used to induce the mouse model of peritonitis and further result in the generation of excessive H_2_O_2_. The deep images of mouse models are captured for 3 min after the injection of P‐CDs (0.3 mg mL^−1^, 0.2 mL) at an early stage of peritonitis (4 h after LPS injection). As shown in **Figure** **5**a,b, the PL intensities are almost unchanged for all the groups due to the structure resistance of P‐CDs toward ROS. In contrast, the CL diagnostic signal for LPS‐treated mice is 2.5‐times higher than that for the control mice (Figure 5c,d). There is no CL when anesthetized mice were treated with intraperitoneal injection of M‐CDs without nanointegration of peroxalate (left) or P‐CDs without loading of M‐CDs (right) after 4 h of LPS treatment, which can confirm that the presence of the isolated LPS cannot arise CL of CDs in vivo (Figure S30, Supporting Information). After the inflamed mice by LPS are remedied with an antioxidant glutathione (GSH), CL signal intensity of the LPS + GSH treated mice shows a 40% reduction. The enhanced‐to‐reduced CL intensity can efficiently, as an inflamed‐to‐normal contrast signal, monitor the variation of inflammatory disease in living animals. The above results demonstrate that NIR CDs can act as a potential CL probe to diagnose and evaluate the state of an illness through sensitive in vivo bioimaging.

## 3 Conclusion

In summary, we have demonstrated efficient NIR emissive CDs‐based CL system via energy transfer from the chemical reaction of peroxalate and H_2_O_2_. With further modification, the CDs can be modified from hydrophilic to hydrophobic. P‐CDs can be produced by combining the CDs, CPPO, and PEG‐b‐PPG‐b‐PEG through the nanoscopic coaggregation. The NIR P‐CDs generate good in vitro and in vivo CL signals in response to H_2_O_2_ with a linear range from 0 to 100 × 10^−9^ m and a low detection limit of 5 × 10^−9^ m. Furthermore, the P‐CDs are proved to be a potential CL probe for bioimaging H_2_O_2_ in the inflammation‐related diseases in living mice. The results reported in this paper may provide a clue for the diagnosis and treatment of inflammation or cancers employing CL CDs as sensors.

## 4 Experimental Section

##### Synthesis and Purification of CDs

An amount of 1 g citric acid and 2 g urea were dispersed in 10 mL of DEF. The mixtures were added into Teflon‐lined stainless autoclave (20 mL). Then the sealed autoclave vessels were placed into an electric oven, which was set at 160 °C and hold for 8 h. The resulting solvents were purified via silica column chromatography using DMF as the eluent, and then the as‐prepared CDs were precipitated with absolute ethyl alcohol and collected by vacuum drying at 60 °C for 1 day. The final products were collected for characterizations and further used.

##### Synthesis and Purification of M‐CDs

An amount of 0.2 g CDs powder, 0.8 g octadecylamine, and 0.1 g mPEG‐NH_2_ were dispersed into 50 mL DMF, where the octadecylamine was used to switch the water−oil soluble of the CDs and mPEG‐NH_2_ was used to increase the cross‐linking of CDs and octadecylamine. After stirring with air flow for 12 h under 60 °C, 10 mL mixture was dispersed in 20 mL CH_2_Cl_2_. With dichloromethane CH_2_Cl_2_ as eluent, the hydrophobic M‐CDs can be extracted from DMF with massive water and kept in CH_2_Cl_2_ solution. The as‐prepared M‐CDs were collected by vacuum drying at 60 °C for 1 day. The final products were collected for characterizations and further used.

##### Synthesis of P‐CDs

PEG‐b‐PPG‐b‐PEG (40 mg), CPPO (4 mg), and different mass of M‐CDs (0.1, 1.0, 1.5, 2, and 3 mg) were dispersed into CH_2_Cl_2_ (5.0 mL) solution. After CH_2_Cl_2_ was evaporated with air flow, the powder was redispersed into 2 mL deionized water and filtered through a 0.22 µm PVDF syringe driven filter (Millipore). The formed P‐CDs suspension was finally concentrated to different concentrations by ultrafiltration and used immediately for experiments.

##### CL QYs of CDs and P‐CDs

The CL QYs of the CDs and P‐CDs were measured using lucigenin with H_2_O_2_ as oxidant with a known QY of 8.3 × 10^−3^ einstein mol^−1^ at pH 11 according to the previous literatures.^[^ ^29^ ^]^ According to the CL spectra and kinetic curves, the CL QYs were calculated according to the following equations:

(1) $$\phi_{\text{CL}}=\frac{Q\times f_{\text{luc}}\times f_{\text{photo}}}{n}(\text{einstein}\;{\text{mol}}^{-1})$$

(2) $$f_{\text{luc}}=\frac{\phi_{\text{luc}}\times n_{\text{luc}}}{Q_{\text{luc}}}$$

(3) $$f_{\text{photo}}=\frac{f(\lambda_{\text{s}})}{f(\lambda_{\text{luc}})}$$

Where ϕ_CL_ is the CL QYs of CDs and P‐CDs, *Q* is the total light emission obtained by integration of emission intensity under time curves. *f*_luc_ is obtained by measuring the emission kinetics of lucigenin reaction performed in standard conditions. *f*_photo_ is obtained from the sensitivity at the emission wavelength (λ_max_ = 475 nm) of the lucigenin standard, *f*(λ_luc_), and the emission of the CDs and P‐CDs, *f*(λ_s_). *n* is the number of moles of lucigenin (*n*_luc_) or the number of moles of CPPO.

##### In Vitro Characterization of P‐CDs

For the quantitative analysis, P‐CDs (10 mg mL^−1^, 1 mL) were placed in EP tubes. After addition of different concentrations (0 × 10^−9^, 5 × 10^−9^, 50 × 10^−9^, 100 × 10^−9^, 1 × 10^−6^, and 5 × 10^−6^ m) of H_2_O_2_, both PL and CL imaging were performed using an IVIS spectrum imaging system. CL images were acquired for 30 s with open filter or emission at 640 ± 10 nm, and fluorescence images were acquired for 0.1 s with excitation of 550 ± 10 nm, and emission at 640 ± 10 nm. The media pH 7.4 was controlled using phosphate buffer saline (PBS) buffer solution.

##### Cytotoxic Evaluation

The PBS solution with different concentrations (0, 0.16, 0.8, 4, 20, 100, and 500 µg mL^−1^) of P‐CDs was used for incubating the Hela cells for 24 h at 37 °C. Then, the cell viability of Hela cell was tested using standard MTT method for assessing the cytotoxicity of the P‐CDs.

##### In Vivo CL Imaging

All animal studies were performed in compliance with the Guide Care and Use of Laboratory Animals proposed by the National Institutes of Health. All procedures and protocols were approved by the Animal Ethics Committee at Zhengzhou University (Zhengzhou, China). For in vivo bioimaging exogenous H_2_O_2_, P‐CDs (10 mg mL^−1^) were mixed with different concentrations (0 × 10^−9^, 5 × 10^−9^, 50 × 10^−9^, and 100 × 10^−9^ m ) of H_2_O_2_, and the P‐CD suspension (0.1 mL) was injected subcutaneous into the dorsal of anaesthetized mice (2% isoflurane in oxygen). The media pH 7.4 was controlled using PBS buffer solution. CL images were obtained with a 3 min acquisition time with open filter or emission at 640 ± 10 nm, and fluorescence images were acquired for 30 s with excitation of 550 ± 10 nm, and emission at 640 ± 10 nm.

To image endogenous H_2_O_2_ in the mouse model of peritonitis, mice (6−8 weeks old) were injected intraperitoneally with LPS (8 mg kg^−1^). For the inhibitor study, mice were treated intraperitoneally 5 min before LPS treatment using 200 mg kg^−1^ GSH. The control mice were treated only using 200 mg kg^−1^ saline. After 4 h, anesthetized mice (2% isoflurane in oxygen) were treated with intraperitoneal injection of P‐CDs (10 mg mL^−1^, 0.2 mL). CL images were captured with a 3 min acquisition time with open filter using the IVIS spectrum imaging system. Fluorescence images were captured with a 0.1 s acquisition time with excitation of 550 ± 10 nm, and emission at 640 ± 10 nm using the IVIS spectrum imaging system.

More details about the characterization of material and data are demonstrated in Supporting Information.

## Conflict of Interest

The authors declare no conflict of interest.

## Supporting information

Supporting Information

## References

[1] B. D'Autréaux , M. B. Toledano , Nat. Rev. Mol. Cell Biol. 2007, 8, 813. 17848967 10.1038/nrm2256

[2] M. Wang , Nat. Rev. Nephrol. 2019, 15, 61. 30514973 10.1038/s41581-018-0090-7

[3] N. Gong , X. Ma , X. Ye , Q. Zhou , X. Chen , X. Tan , S. Yao , S. Huo , T. Zhang , S. Chen , X. Teng , X. Hu , J. Yu , Y. Gan , H. Jiang , J. Li , X. Liang , Nat. Nanotechnol. 2019, 14, 379. 30778211 10.1038/s41565-019-0373-6

[4] Ö. Canli , A. M. Nicolas , J. Gupta , F. Finkelmeier , O. Goncharova , M. Pesic , T. Neumann , D. Horst , M. Löwer , U. Sahin , F. R. Greten , Cancer Cell 2017, 32, 869. 29232557 10.1016/j.ccell.2017.11.004

[5] A. J. Shuhendler , K. Pu , L. Cui , J. P. Uetrecht , J. Rao , Nat. Biotechnol. 2014, 32, 373. 24658645 10.1038/nbt.2838 PMC4070437

[6] W. Dröge , Physiol. Rev. 2002, 82, 47. 11773609 10.1152/physrev.00018.2001

[7] H. Blaser , C. Dostert , T. W. Mak , D. Brenner , Trends Cell Biol. 2016, 26, 249. 26791157 10.1016/j.tcb.2015.12.002

[8] C. Tapeinos , A. Pandit , Adv. Mater. 2016, 28, 5553. 27184711 10.1002/adma.201505376

[9] S. Bhattacharya , R. Sarkar , S. Nandi , A. Porgador , R. Jelinek , Anal. Chem. 2017, 89, 830. 27991760 10.1021/acs.analchem.6b03749

[10] Y. Chen , A. J. H. Spiering , S. Karthikeyan , G. W. M. Peters , E. W. Meijer , R. P. Sijbesma , Nat. Chem. 2012, 4, 559. 22717441 10.1038/nchem.1358

[11] Y. Liu , W. Shen , Q. Li , J. Shu , L. Gao , M. Ma , W. Wang , H. Cui , Nat. Commun. 2017, 8, 1003. 29042537 10.1038/s41467-017-01101-6 PMC5645356

[12] S. Kwak , J. P. Giraldo , M. H. Wong , V. B. Koman , T. T. S. Lew , J. Ell , M. C. Weidman , R. M. Sinclair , M. P. Landry , W. A. Tisdale , M. S. Strano , Nano Lett. 2017, 17, 7951. 29148804 10.1021/acs.nanolett.7b04369

[13] Y. Lee , C. Lim , A. Singh , J. Koh , J. Kim , I. C. Kwon , S. Kim , ACS Nano 2012, 6, 6759. 22747065 10.1021/nn3014905

[14] X. Zhen , C. Zhang , C. Xie , Q. Miao , K. L. Lim , K. Pu , ACS Nano 2016, 10, 6400. 27299477 10.1021/acsnano.6b02908

[15] Q. Miao , K. Pu , Adv. Mater. 2018, 30, 1801778. 10.1002/adma.201801778 30058244

[16] F. Hu , S. Xu , B. Liu , Adv. Mater. 2018, 30, 1801350. 10.1002/adma.201801350 30066341

[17] O. Green , S. Gnaim , R. Blau , A. Eldar‐Boock , R. Satchi‐Fainaro , D. Shabat , J. Am. Chem. Soc. 2017, 139, 13243. 28853880 10.1021/jacs.7b08446

[18] C. Zhu , Y. Fu , C. Liu , Y. Liu , L. Hu , J. Liu , I. Bello , H. Li , N. Liu , S. Guo , H. Huang , Y. Lifshitz , S. Lee , Z. Kang , Adv. Mater. 2017, 29, 1701399. 10.1002/adma.201701399 28640515

[19] Z. Wang , F. Yuan , X. Li , Y. Li , H. Zhong , L. Fan , S. Yang , Adv. Mater. 2017, 29, 1702910. 10.1002/adma.201702910 28758696

[20] X. Miao , D. Qu , D. Yang , B. Nie , Y. Zhao , H. Fan , Z. Sun , Adv. Mater. 2018, 30, 1704740. 10.1002/adma.201704740 29178388

[21] F. Yuan , Z. Wang , X. Li , Y. Li , Z. Tan , L. Fan , S. Yang , Adv. Mater. 2017, 29, 1604436. 10.1002/adma.201604436 27879013

[22] S. Bhattacharya , R. S. Phatake , S. Nabha Barnea , N. Zerby , J. Zhu , R. Shikler , N. G. Lemcoff , R. Jelinek , ACS Nano 2019, 13, 7396. 30615415 10.1021/acsnano.8b07087

[23] S. Qu , X. Liu , X. Guo , M. Chu , L. Zhang , D. Shen , Adv. Funct. Mater. 2014, 24, 2689.

[24] F. Wang , Z. Xie , H. Zhang , C. Liu , Y. Zhang , Adv. Funct. Mater. 2011, 21, 1027.

[25] S. N. Baker , G. A. Baker , Angew. Chem., Int. Ed. 2010, 49, 6726. 10.1002/anie.200906623 20687055

[26] K. K. Liu , S. Y. Song , L. Z. Sui , S. X. Wu , P. T. Jing , R. Q. Wang , Q. Y. Li , G. R. Wu , Z. Z. Zhang , K. J. Yuan , C. X. Shan , Adv. Sci. 2019, 6, 1900766. 10.1002/advs.201900766 PMC6724478 31508282

[27] S. Bhattacharya , R. Sarkar , B. Chakraborty , A. Porgador , R. Jelinek , ACS Sens. 2017, 2, 1215. 28770991 10.1021/acssensors.7b00356

[28] N. Shauloff , S. Bhattacharya , R. Jelinek , Carbon 2019, 152, 363.

[29] C. L. Shen , Q. Lou , C. F. Lv , J. H. Zang , S. N. Qu , L. Dong , C. X. Shan , Adv. Sci. 2019, 6, 1802331. 10.1002/advs.201802331 PMC6548985 31179212

[30] S. Hu , A. Trinchi , P. Atkin , I. Cole , Angew. Chem., Int. Ed. 2015, 54, 2970. 10.1002/anie.201411004 25589468

[31] S. Zhu , Q. Meng , L. Wang , J. Zhang , Y. Song , H. Jin , K. Zhang , H. Sun , H. Wang , B. Yang , Angew. Chem., Int. Ed. 2013, 52, 3953. 10.1002/anie.201300519 23450679

[32] S. Lu , L. Sui , J. Liu , S. Zhu , A. Chen , M. Jin , B. Yang , Adv. Mater. 2017, 29, 1603443. 10.1002/adma.201603443 28195369

[33] K. Holá , M. Sudolská , S. Kalytchuk , D. Nachtigallová , A. L. Rogach , M. Otyepka , R. Zbořil , ACS Nano 2017, 11, 12402. 29136460 10.1021/acsnano.7b06399

[34] H. Yu , Y. Xue , Y. Li , Adv. Mater. 2019, 31, 1803101. 10.1002/adma.201803101 31119816

[35] J. Tang , B. Kong , H. Wu , M. Xu , Y. Wang , Y. Wang , D. Zhao , G. Zheng , Adv. Mater. 2013, 25, 6569. 23996326 10.1002/adma.201303124

[36] A. Zhu , Q. Qu , X. Shao , B. Kong , Y. Tian , Angew. Chem., Int. Ed. 2012, 51, 7185. 10.1002/anie.201109089 22407813

[37] S. Lu , G. Xiao , L. Sui , T. Feng , X. Yong , S. Zhu , B. Li , Z. Liu , B. Zou , M. Jin , J. S. Tse , H. Yan , B. Yang , Angew. Chem., Int. Ed. 2017, 56, 6187. 10.1002/anie.201700757 28378520

[38] W. Li , Z. Zhang , B. Kong , S. Feng , J. Wang , L. Wang , J. Yang , F. Zhang , P. Wu , D. Zhao , Angew. Chem., Int. Ed. 2013, 52, 8151. 10.1002/anie.201303927 23788215

[39] J. Liu , N. Wang , Y. Yu , Y. Yan , H. Zhang , J. Li , J. Yu , Sci. Adv. 2017, 3, e1603171. 28560347 10.1126/sciadv.1603171 PMC5446214

[40] D. Qu , M. Zheng , J. Li , Z. Xie , Z. Sun , Light: Sci. Appl. 2015, 4, e364.

[41] R. Sekiya , Y. Uemura , H. Murakami , T. Haino , Angew. Chem., Int. Ed. 2014, 53, 5619. 10.1002/anie.201311248 24711343

[42] Z. Tian , X. Zhang , D. Li , D. Zhou , P. Jing , D. Shen , S. Qu , R. Zboril , A. L. Rogach , Adv. Opt. Mater. 2017, 5, 1700416.

