# Supplementary material for: Near‐Infrared Chemiluminescent Carbon Nanodots and Their Application in Reactive Oxygen Species Bioimaging
Source: Adv Sci (Weinh). 2020 Mar 9;7(8):1903525. doi: 10.1002/advs.201903525 (PMC7175254; doi:10.1002/advs.201903525)
Supplement: Supplementary file 1 — Supporting Information [file ADVS-7-1903525-s001.pdf]

Copyright WILEY-VCH Verlag GmbH & Co. KGaA, 69469 Weinheim, Germany, 2020.

ADVANCED SCIENCE
Open Access

# Supporting Information

for *Adv. Sci.,* DOI: 10.1002/advs.201903525

# Near-Infrared Chemiluminescent Carbon Nanodots and Their Application in Reactive Oxygen Species Bioimaging

*Cheng-Long Shen, Qing Lou,* Jin-Hao Zang, Kai-Kai Liu, Song-Nan Qu, Lin Dong, and Chong-Xin Shan**

Copyright WILEY-VCH Verlag GmbH & Co. KGaA, 69469 Weinheim, Germany, 2020.

**Supporting Information**

**Near-Infrared Chemiluminescent Carbon Nanodots and Their Application in Reactive Oxygen Species Bioimaging**

*Cheng-Long Shen, Qing Lou*, Jin-Hao Zang, Kai-Kai Liu, Song-Nan Qu, Lin Dong, Chong-Xin Shan**

C. L. Shen, Dr. Q. Lou, J. H. Zang, Dr. K. K. Liu, Prof. L. Dong, Prof. C. X. Shan
Henan Key Laboratory of Diamond Optoelectronic Materials and Devices, Key Laboratory of Materials Physics, Ministry of Education, School of Physics and Microelectronics, Zhengzhou University, Zhengzhou, 450052, China
E-mail: louqing1986@zzu.edu.cn (Q. Lou), cxshan@zzu.edu.cn (C. X. Shan)
Prof. S. N. Qu,
Joint Key Laboratory of the Ministry of Education, Institute of Applied Physics and Materials Engineering, University of Macau, Macau, 999078, China

## EXPERIMENTAL SECTION

### Materials.

All the chemicals and solvents were used without further purification.

### Characterization.

The surface morphology of the three CDs was characterized by a field emission transmission electron microscope (HRTEM, JEOL JSM-IT100). The absorption spectra were measured on a Hitachi U-3900 UV-VIS-NIR spectrophotometer. The fluorescence spectra were measured by a spectrofluorometer (Hitachi F-7000). The fluorescence decay curves were measured by a Horiba FL-322 spectrometer using a 405 nm NanoLED monitoring the emission at 642 nm, respectively. The absolute PL QYs were also measured by a Horiba FL-322 spectrometer with a 365 nm NanoLED as excitation source. X-ray photoelectron spectroscopy (XPS) was measured on a Kratos AXIS HIS 165 spectrometer with a monochromatized Al KR X-ray source (1486.7 eV). The Fourier transform infrared spectra (FT-IR) of the CDs were recorded

on a Bio-Rad Excalibur spectrometer (Bruker vector 22). The X-ray diffractometer (Panalytical X' Pert Pro) using Cu $k_\alpha$ as the irradiation source was used to obtain the XRD patterns. Raman spectra were carried on a Raman spectrometer (Renishaw inVia). The optical and chemiluminiscence (CL) images were obtained using a Nikon D600 digital camera. The maximal CL luminance of the CDs was measured by using a system comprising a photometer (Minolta Luminance Meter LS-110) during the continuous process of CL. The information was printed on an ink cartridge printer (Epson L310).

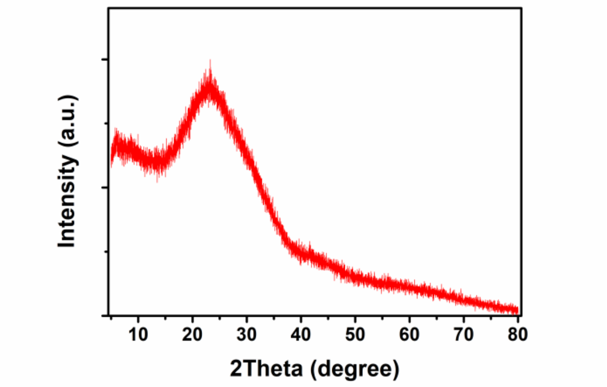


**Figure S1.** The XRD pattern of the CDs.

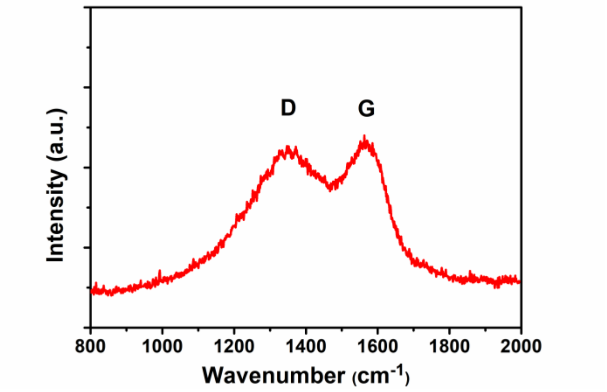


**Figure S2.** The Raman spectra of the CDs.

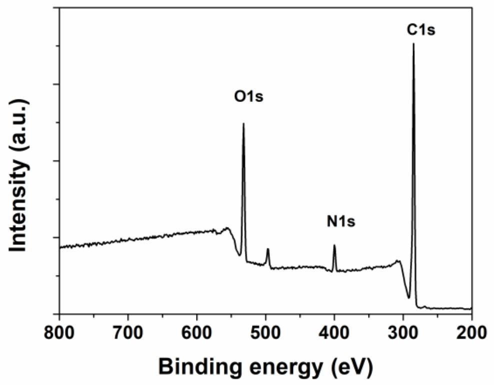


**Figure S3.** The Full survey XPS spectra of the CDs.

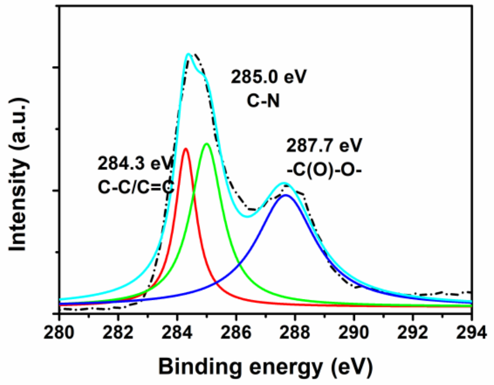


**Figure S4.** The C1s spectra of the CDs.

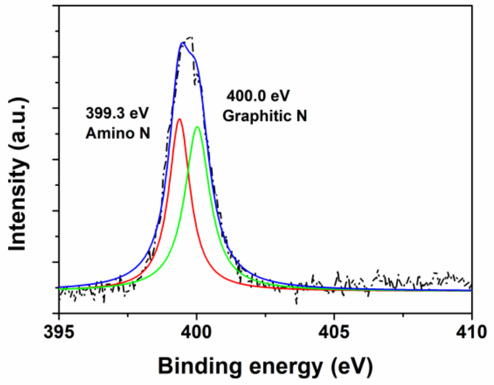


**Figure S5.** The N1s spectra of the CDs.

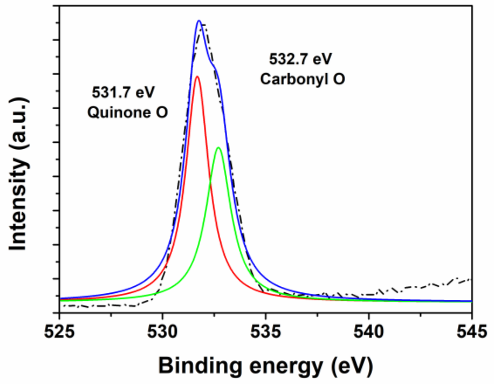


**Figure S6.** The O1s spectra of the CDs.

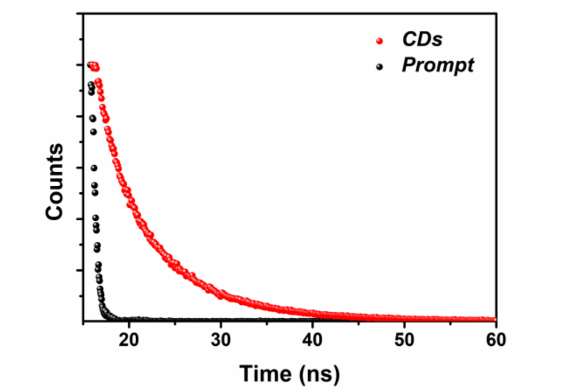


**Figure S7.**Time-resolved decay spectra of the CDs in aqueous solution.

**Chemiluminescence measurements.**

The CL spectra were measured by the Hitachi F-7000 spectrofluorometer with the excitation light source off. 1 mL lucigenin ($5.8\times10^{-5}$ M) aqueous soulution at PH=11 were injected into the 1 mL $H_2O_2$ (0.1 M) aqueous solution and the CL spectra was measure by the spectrofluorometer. The CL kinetic curves were also measured by the F-7000 spectrofluorometer. On the same condition, 0.5 mL CDs (3 mg $mL^{-1}$) ethyl alcohol solution were injected into the mixture of 1 mL CPPO (0.2 mM) ethyl acetate solution and 1 mL $H_2O_2$ (0.2 M) ethyl alcohol solution and the CL spectra was measure by the spectrofluorometer. The CL kinetic curves were also measured by the F-7000 spectrofluorometer.

**Chemiluminescence quantum yield (CL QYs) of the CDs.**

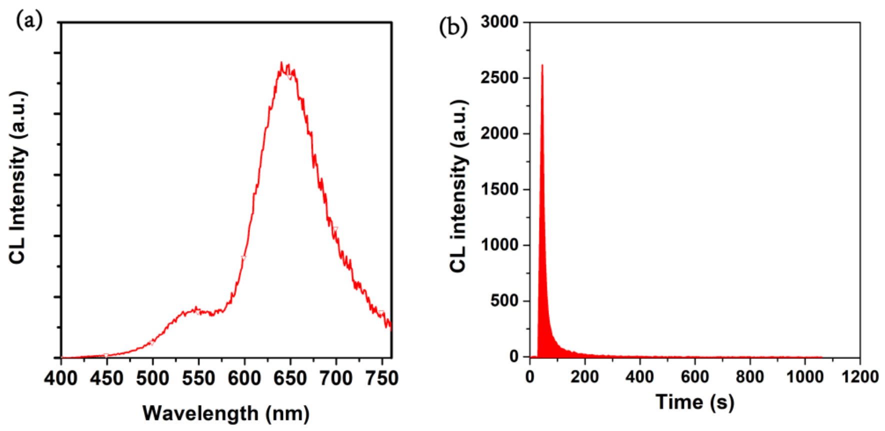


**Figure S8.** The CL spectra (a) and decay curves (b) of the CDs.

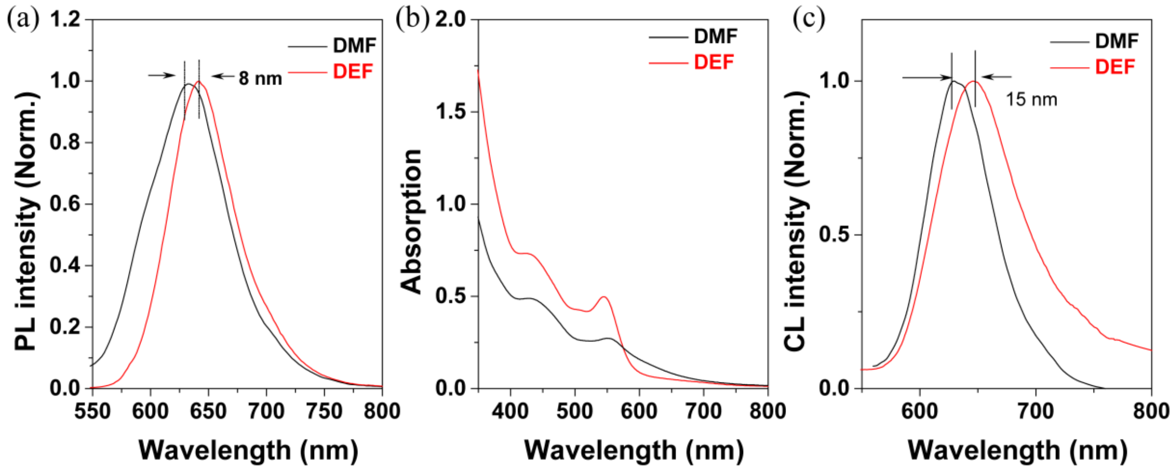


**Figure S9.** The PL (a), absorbance (b), and CL (c) data o the CDs synthesized by DMF and DEF.

**Table S1.** PL and CL characteristics of the CDs synthesized by DMF and DEF.

| Samples | $\lambda_{em}$ [a] (nm) | $\tau$ [c] (ns) | $\lambda_{em}$ [d] (nm) | $\Phi_C$ [e] (einsteins $mol^{-1}$) |
|---|---|---|---|---|
| $CD_{DMF}$ | 634 | 5.2 | 631 | $9.32\times10^{-3}$ |
| $CD_{DEF}$ | 642 | 5.9 | 647 | $9.98\times10^{-3}$ |

[a] PL maximum peak; [b] PL lifetime; [c] CL maximum peak; [d] CL quantum yield.

**Note :** It can be found the CDs synthesized by DEF (N,N-diethylformamide) compared with DMF (N,N-dimethylformamide) exhibit 8 nm peak shift in PL and 15 nm peak shift in CL. And the corresponding exciton absorption peak has a blue shift. Moreover, the CL induced by the CDs synthesized by DEF can extend to the ~800 nm NIR region, which is beneficial to be applied in NIR bioimaging. In our previous report, we have demonstrated the solvent on the synthesis can tune the degree of graphitization and size of conjugated $sp^2$-domains of CDs, which can result in different PL and CL emission (see *Adv. Sci.* **2019**, *6*, 1802331). The changing solvent plays an important role in regulating PL of CDs, which has been verified by other reports (see *Sci. Adv.* **2017**, *3*, e1603171; *Light-Sci. Appl.* **2015**, *4*, e364; *Adv. Mater.* **2018,** *30*, 1704740; *Adv. Optical Mater.* **2017**, *5*, 1700416 and *Carbon* **2018**, *136*,

359). In this work, we chose DEF rather than DMF as the solvent to synthesis CDs. DEF and DMF, as polar aprotic solvents, cannot donate hydrogen in the reaction. Relative large effective conjugation length of CDs is a result of dehydration reaction happened between citric acid and intra-molecules. In addition, intra-molecules with longer carbon chains (ethyl group from DEF vs. methyl group from DMF) can exacerbate dehydration reaction and will increase the conjugation degree of CDs (see *Light-Sci. Appl.* **2015**, *4*, e364 and *Adv. Sci.* **2019**, *6*, 1802331). Thus, emission red-shift of CDs will appear and more PL emission is extended to the NIR region (> 650 nm) in spite of only an 8 nm peak shift in PL due to the changing solvents from DMF to DEF.

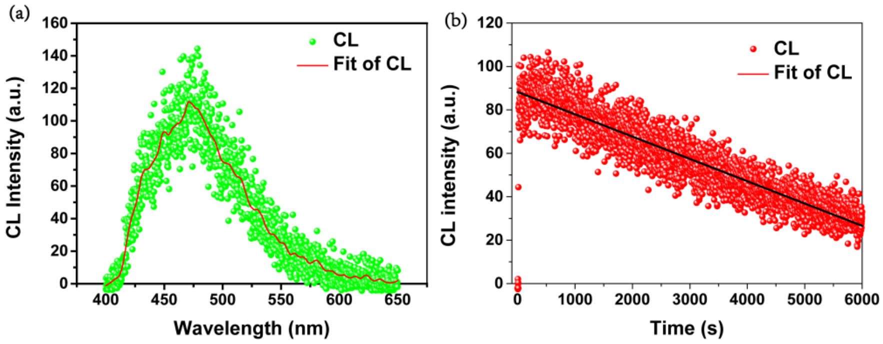


**Figure S10.** a,b) The CL spectra (a) and decay curves (b) of lucigenin.

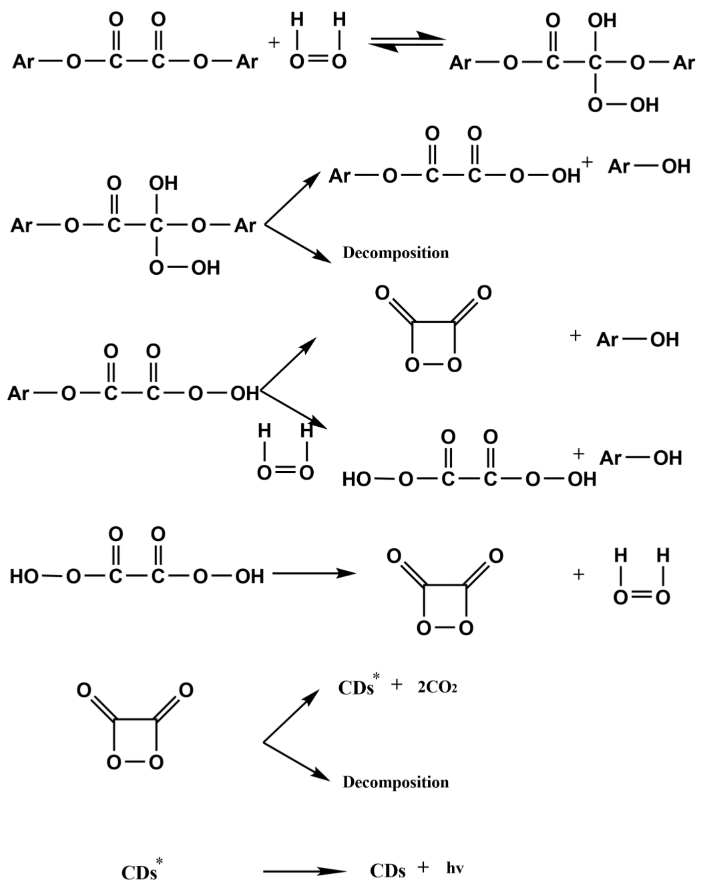


**Figure S11.** Schematic illustration of the CL process of the CDs.

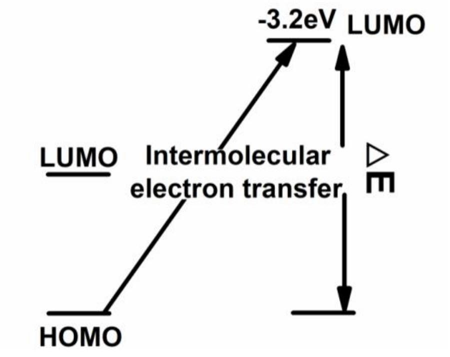


**Figure S12.** Schematic illustration of the CIEEL mechanism of the CDs.

**Chemiluminescence intensity measurement of the CDs.**

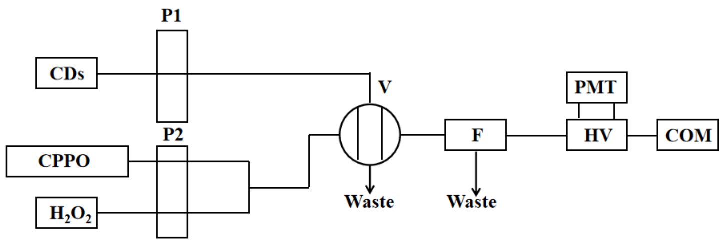


**Figure S13.** Schematic illustration of the structure of flow-injection CL analysis device.

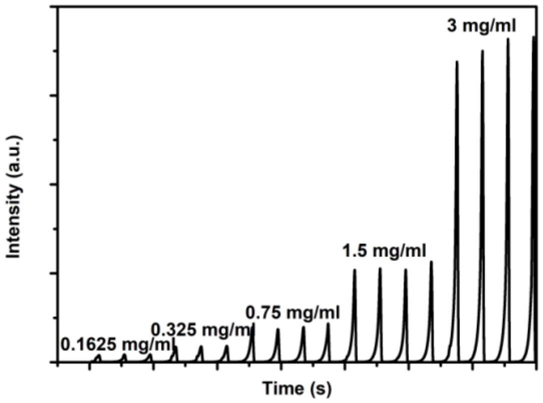


**Figure S14.** The CL intensity curve for different concentration of the CDs.

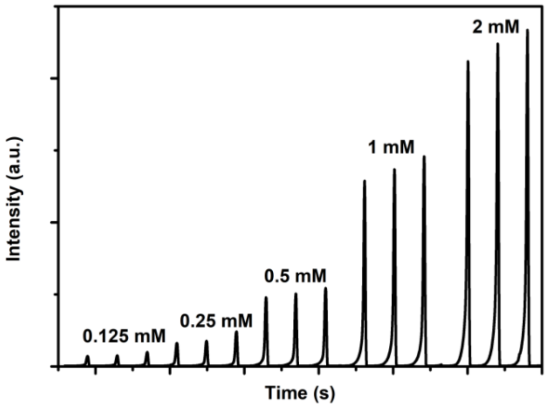


**Figure S15.** The CL intensity curve for concentration of CPPO.

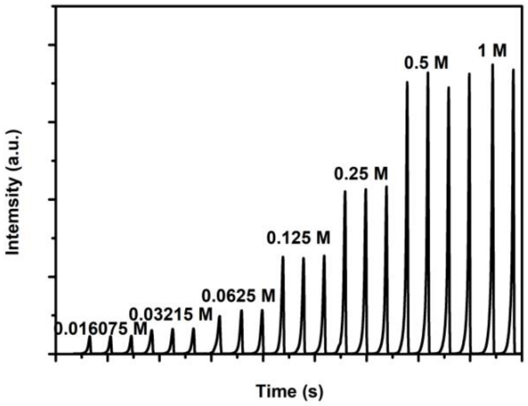


**Figure S16.** The CL intensity curve for different concentration of $H_2O_2$.

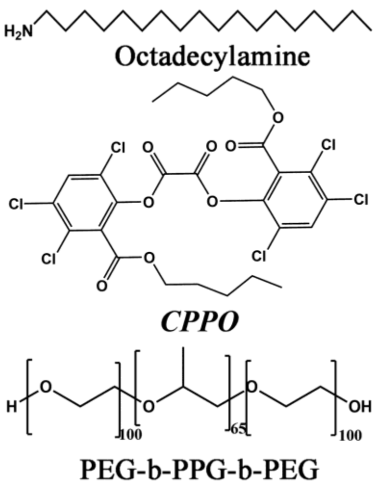


**Figure S17.** Chemical structure of octadecylamine, CPPO and PEG-b-PPG-b-PEG.

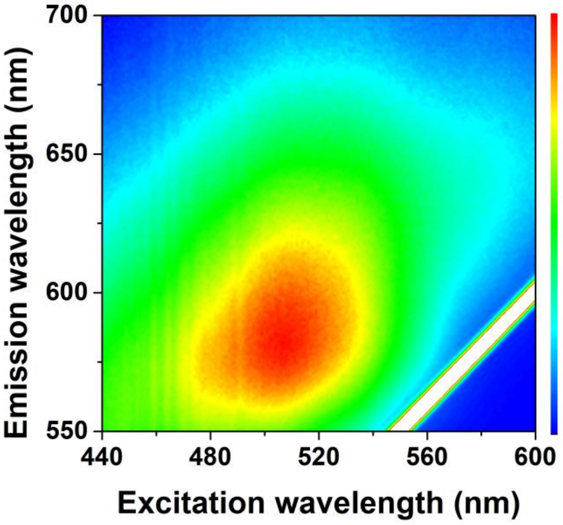


**Figure S18.** The excitation-emission matrix for the CDs-M.

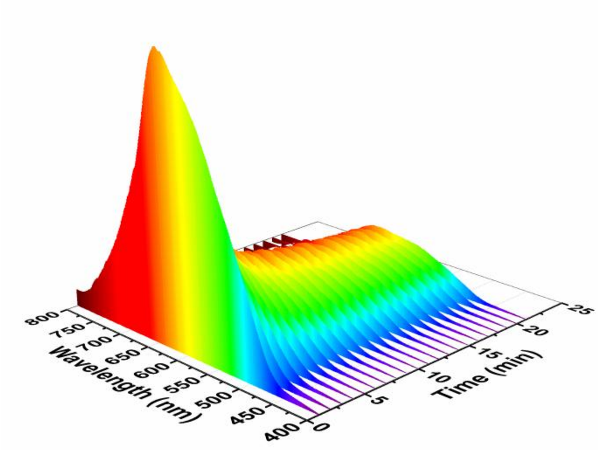


**Figure S19.** The CL decay spectra of the CL from the M-CDs.

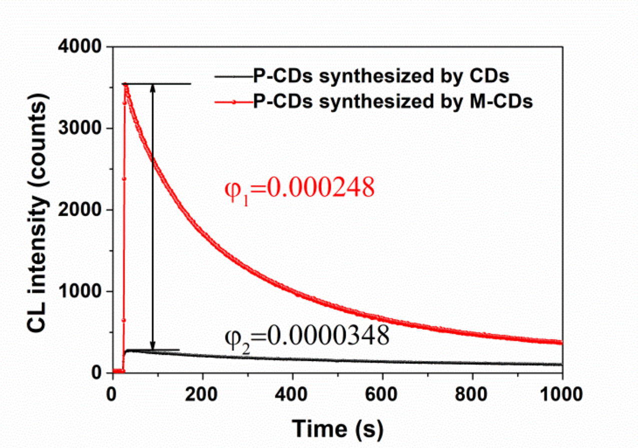


**Figure S20.**The CL decay of the P-CDs synthesized with the CDs and CDs-M.

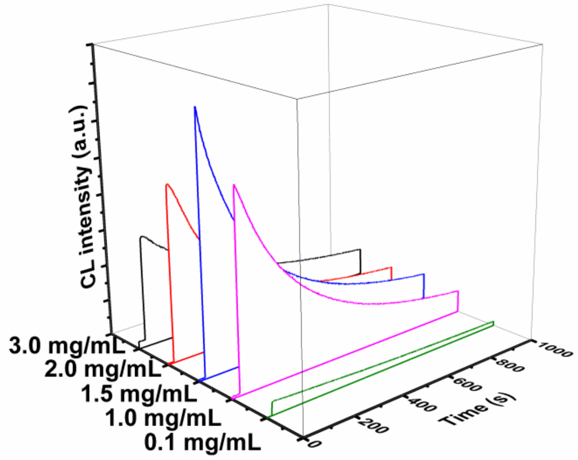


**Figure S21.** The CL intensity decrease with time for the P-CDs synthesized with different concentration of CDs-M.

**Note:** The CL quantum yield (CL QY) has optimized by changing the loading weight of CDs in the p-CDs. Amongst them, the maximum CL QY of the P-CDs is loaded with 2 mg CDs. Hence, we employed the concentration of CDs in the P-CDs as bioimaging probes for in vitro and in vivo detecting $H_2O_2$.

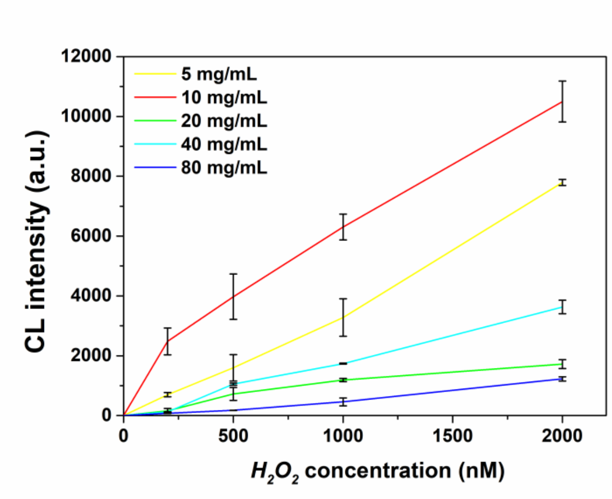

**Figure S22** The CL intensity *vs.* $H_2O_2$ concentration induced by different concentration of P-CDs in the CL analysis instrument.

**Note:** The CL intensity *vs.* $H_2O_2$ concentration induced by different concentration of P-CDs has been also measured with the CL analysis instrument. As shown in Figure S22, the P-CDs (10 mg/mL) have the highest CL intensity value with the $H_2O_2$ concentration ranging from 0 to 2000 nM. With the further increase in the concentration of P-CDs, the CL intensity decreases, which may induced by the aggregation induced quenching (AIQ) effect due to the strong interaction of CDs. Hence, we select the 10 mg/mL P-CDs loaded with 2 mg CDs as bioimaging probes for the in vivo CL imaging.

**Results**

| | | | Size (d.n... | % Intensity: | St Dev (d.n... |
|---|---|---|---|---|---|
| Z-Average (d.nm): | 35.93 | Peak 1: | 39.76 | 97.0 | 16.10 |
| PdI: | 0.229 | Peak 2: | 4814 | 3.0 | 721.4 |
| Intercept: | 0.906 | Peak 3: | 0.000 | 0.0 | 0.000 |
| Result quality | Good | | | | |

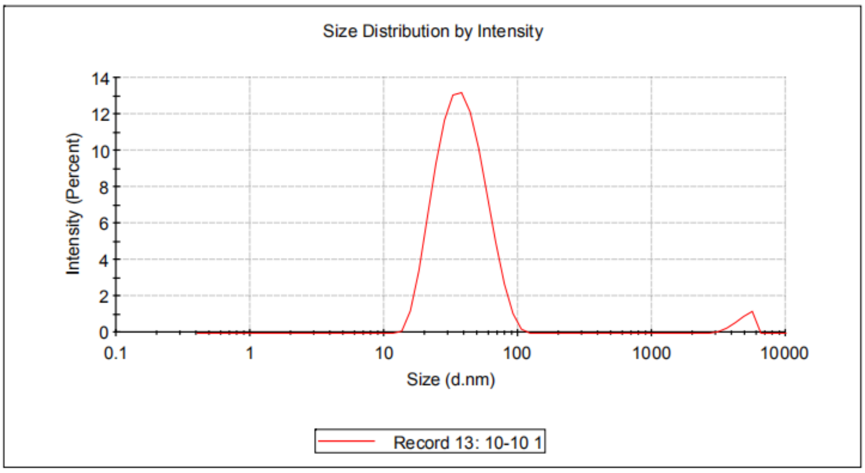


**Figure R23.** The DLS distribution of the P-CDs (Intensity vs. Size).

**Results**

| | | | Size (d.nm): | % Volume: | St Dev (d.nm): |
|---|---|---|---|---|---|
| Z-Average (d.nm): | 35.93 | Peak 1: | 26.51 | 99.8 | 10.21 |
| PdI: | 0.229 | Peak 2: | 5093 | 0.2 | 827.6 |
| Intercept: | 0.906 | Peak 3: | 0.000 | 0.0 | 0.000 |
| Result quality | Good | | | | |

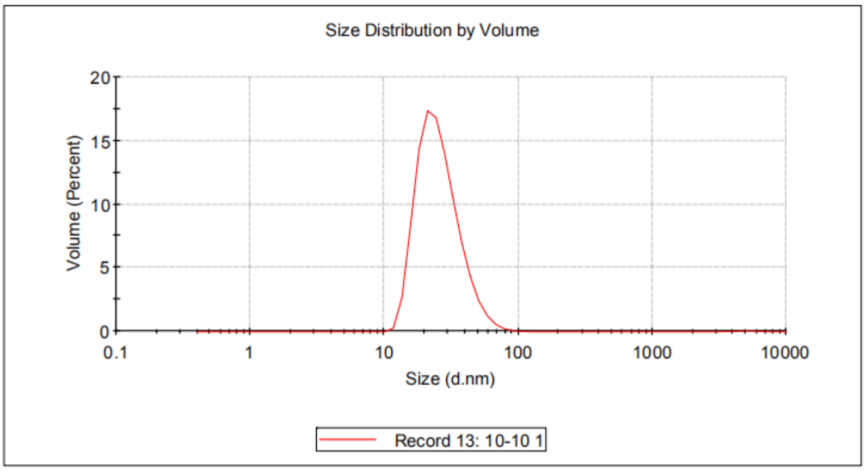


**Figure R24.** The DLS distribution of the P-CDs (Volume vs. Size).

**Note:** The rest of the DLS spectra (Intensity, Volume) have be provided according the reviewer's suggestion. The intensity-weighted hydrodynamic size from the DLS is about 40 nm, which is the same with the number-weighted hydrodynamic diameter of P-CDs. Nevertheless, the volume-weighted hydrodynamic size is about 27 nm, which is a little smaller than the number-weighted results probably due to the concentrated number distribution in the small size region.

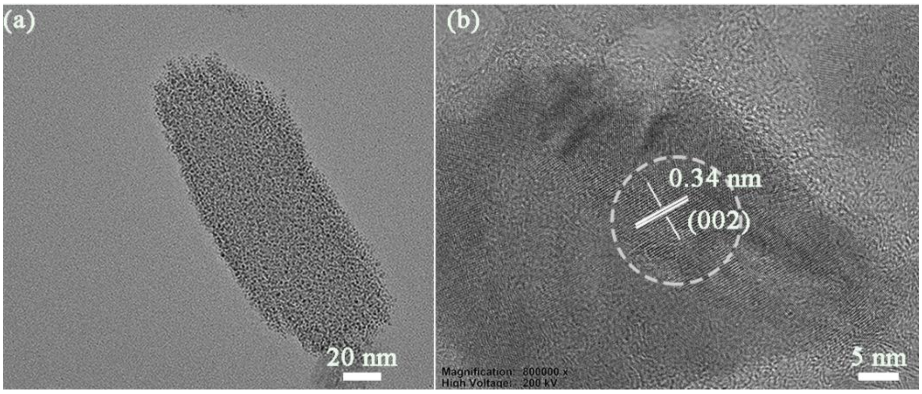


**Figure S25.** The TEM (a) and HRTEM (b) images of P-CDs synthesized with CDs-M

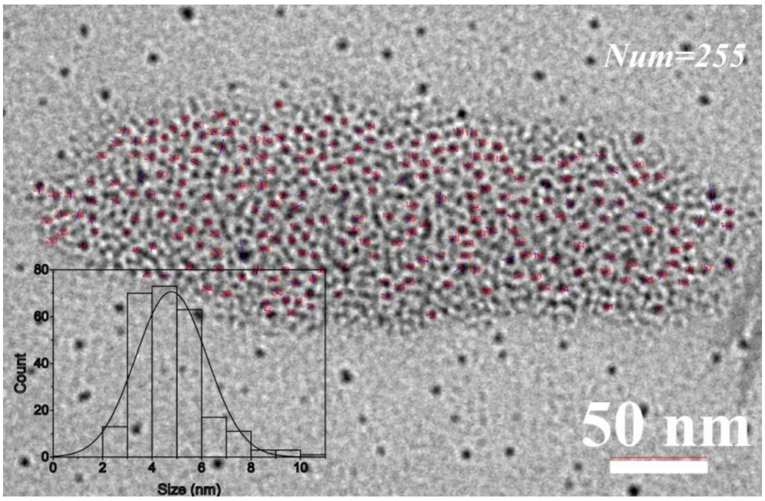


**Figure S26.** HRTEM images of P-CDs (inset: the size distribution of the CDs-M).

**Note:** As an example, we have estimated that one P-CD with diameter of about 100 nm can contain more than 255 M-CDs.

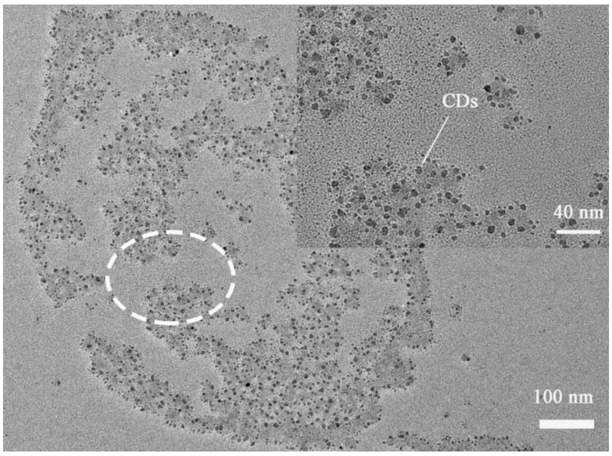


**Figure S27.** The TEM images of the P-CDs synthesized with CDs.

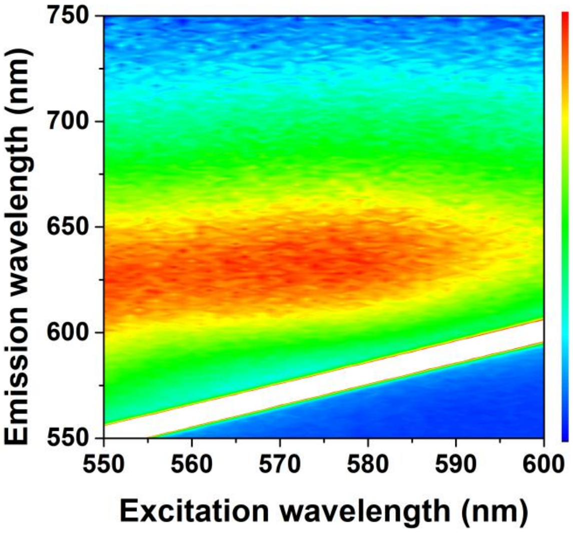


**Figure S28.** The excitation-emission matrix for the P-CDs synthesized with CDs-M.

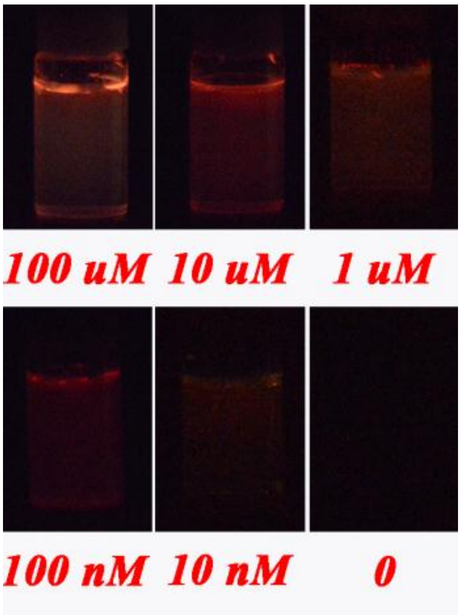


**Figure S29.** The photograph for the P-CDs with different concentration of $H_2O_2$ captured with 30 s.

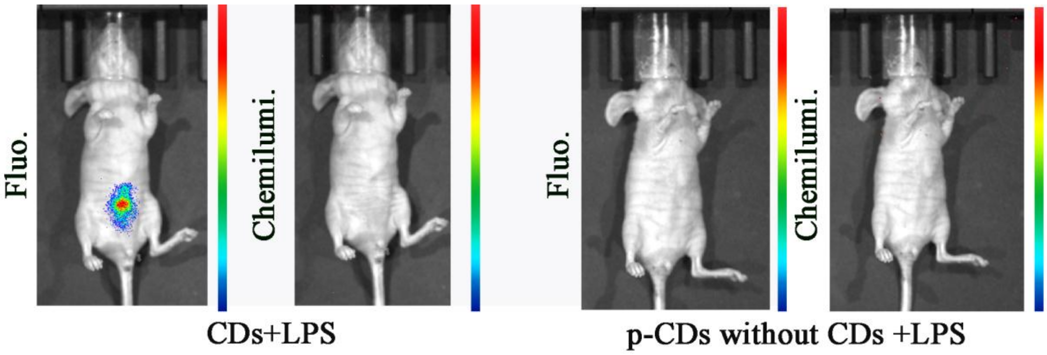


**Figure S30.** In vivo PL and CL images of CDs without nanointegration of peroxalate (left) and p-CDs synthesized without loading of CDs (right) for the mice intraperitoneally treated with LPS.

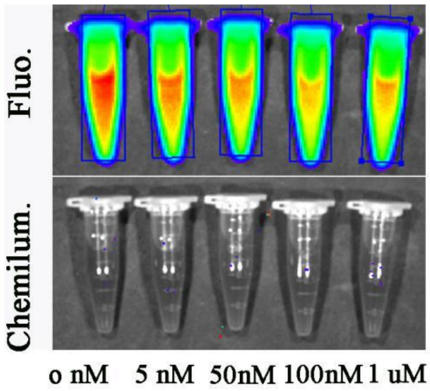


**Figure S31.** In vitro PL and CL bioimaging of the NIR-CDs without conjugation to peroxalate in different concentration of $H_2O_2$.

Note: It can be found the NIR-CDs without conjugation to peroxalate exhibit similar PL properties. While, there are no CL to $H_2O_2$ concentration using the NIR-CDs without conjugation to peroxalate.

**Table S2** The chemiluminescence property of different CDs in previous reports.

| | PL peak [nm] | CL peak [nm] | CL QY [einsteins mol$^{-1}$] | CL system | Refs |
|---|---|---|---|---|---|
| CDs | 642 | 635 | $9.98\times10^{-3}$ | CPPO-CDs-$H_2O_2$ | This work |
| b-CDs | 476 | 468 | $6.60\times10^{-4}$ | CPPO-CDs-$H_2O_2$ | [1] |
| g-CDs | 543 | 526 | $2.52\times10^{-3}$ | CPPO-CDs-$H_2O_2$ | [1] |
| r-CDs | 634 | 631 | $9.32\times10^{-3}$ | CPPO-CDs-$H_2O_2$ | [1] |
| CDs | 454 nm | - | - | CDs-$K_2S_2O_8$-TEA | [2] |
| Gly-CQDs | 485 nm | 450 nm | - | luminol–$KMnO_4$–CQDs | [3] |
| CDs | 410 nm | 425 nm | - | CDs-luminol | [4] |
| CDs | 450 nm | 500 nm | - | Ce(IV)-$Na_2S_2O_3$-CDs | [5] |
| CDs | 450 nm | 500 nm | - | CDs/$K_3Fe(CN)_6$ | [6] |
| CTAB@CD | 410 nm | 530 nm | - | CTAB@CD-Co(II)–$H_2O_2$–$OH^-$ | [7] |
| CDs | 450 nm | 510 nm | - | CDs-$NaNO_2$-$H_2O_2$ | [8] |
| CDs | 525 nm | 555 nm | - | CDs-NaOH | [9] |
| CDs | 520nm | 440 nm/ 610 nm | - | CDs-$KMnO_4$ | [10] |
| r-CDs | 440 nm | 440 nm | - | r-CDs-$KMnO_4$ | [10] |
| CDs | 360 nm | 490 nm | - | CDs-$H_2O_2$-$HSO_3$ | [11] |
| CDs | 450 nm | 500nm/ 650 nm | - | CDs–$KMnO_4$ | [12] |
| CDs | 450 nm | 500 nm | - | CDs–cerium(IV) | [12] |

| | | | | | |
|---|---|---|---|---|---|
| FPOA NPs | 578 | 556 | $4.4 \times 10^{-3}$ | FPOA NPs | [13] |
| SPN-PFO | 436nm | 436nm | $8.16 \times 10^{-5}$ | SPN-PFO-$H_2O_2$ | [14 |
| SPN-PFVA | 566 nm | 566 nm | $7.88 \times 10^{-4}$ | SPN-PFVA-$H_2O_2$ | [14] |
| SPN-PFPV | 507 nm | 507 nm | $2.30 \times 10^{-2}$ | SPN-PFPV-$H_2O_2$ | [14] |
| SPN-PFBT | 534 nm | 534 nm | $2.18 \times 10^{-5}$ | SPN-PFBT-$H_2O_2$ | [14] |
| SPN-PFODBT, | 698 nm | 698 nm | $2.07 \times 10^{-4}$ | SPN-PFODBT-$H_2O_2$ | [14] |
| 6.0-nm gold colloids | 415 nm | 415 nm | $(2.8 \pm 0.3) \times 10^{-5}$ | CPPO-Au NPs-$H_2O_2$ | [15] |
| Cy5 | 698nm | 701nm | $7.5 \times 10^{-4}$ | CPPO-CDs-$H_2O_2$ | [16] |

**Table S3.** Comparison of analytical performance of different CD-based nanosensors for $H_2O_2$ determination.

| Sensing materials | Method | Linear range | LOD[a)] | Ref. |
|---|---|---|---|---|
| CDs | Chemiluminescence | 5-100 nM | 5 nM | This work |
| CQDs | Fluorescence | 0.5-50 uM | 0.2 uM | [17] |
| B-CQDs | Fluorescence | 0.1-10 mM | ---- | [18] |
| GQDs | Fluorescence | 2-300 uM | 0.3 uM | [19] |
| Carbon dots | Fluorescence andColorimetric | 0.05-0.5 M | 45 mM | [20] |
| GDs | Colorimetric | 0.01-10 uM | 10 nM | [24] |
| C-dots/$V_2O_5$ | Colorimetric | 0.5-520 μM | 0.5 uM | [22] |
| C-dots/Pt | Colorimetric | 2.5-1000 uM | 0.8uM | [23] |
| FeOOH/Carbon sheet | Colorimetric | 5-19 uM | 5 nM | [24] |
| GQDs | Colorimetric | 0.02-0.6 mM | 6 uM | [25] |
| GQDs/AgNPs | Colorimetric | ---- | 33 nM | [26] |
| N-GQDs | Colorimetric | 20-1170 uM | 5.3 uM | [27] |
| r-CDs | Colorimetric | 0.01-0.1 mM | ---- | [28] |

| | | | | |
|---|---|---|---|---|
| CQDs | Colorimetric | 1-100 uM | 0.2 μM | [29] |

[a] Limit of detection.


[1] C. L. Shen, Q. Lou, C. F. Lv, J. H. Zang, S. N. Qu, L. Dong, C. X. Shan, *Adv. Sci.* **2019**, *6*, 1802331.

[2] H. Zhang, X. Zhang, and S. Dong. *Anal. Chem.* **2015**, *87*, 11167.

[3] Z. Yan, Y. Yu, J. Chen, *Anal. Methods* **2015**, *7*, 1133.

[4] Y. Guo, B. Li, *Carbon* **2015**, *82*, 459.

[5] M. Amjadi, J. L. Manzoori, T. Hallaj, M. H. Sorouraddin, *Microchim. Acta* **2014**, *181*, 671.

[6] M. Amjadi, J. L. Manzoori, T. Hallaj, M. H. Sorouraddin. S*pectrochim.* A*cta* **2014**, *122*, 715.

[7] J. Shi, C. Lu, D. Yan, L. Ma, *Biosens. Bioelectro.* **2013**, *45*, 58.

[8] Z. Lin, W. Xue, H. Chen, J. Lin, *Anal. Chem.* **2011**, *83*, 8245.

[9] L. Zhao, F. Di, D. Wang, L. Guo, Y. Yang, B. Wan, H. Zhang, *Nanoscale* **2013**, *5*, 2655.

[10] P. Teng, J. Xie, Y. Long, X. Huang, R. Zhu, X. Wang, L. Liang, Y. Huang, H. Zheng, *J. Lumin.* **2014**, *146*, 464.

[11] W. Xue, Z. Lin, H. Chen, C. Lu, J. M. Lin. *J. Phys. Chem. C* **2011**, *115*, 21707.

[12] Z. Lin, W. Xue, H. Chen, J. Lin, *Chem. Commun.* **2012**, *48*, 1051.

[13]Y. Lee, C. Lim, A. Singh, J. Koh, J. Kim, I. C. Kwon, S. Kim, *ACS Nano* **2012**, *6*, 6759.

[14] X. Zhen, C. Zhang, C. Xie, Q. Miao, K. L. Lim, K. Pu, *ACS Nano* **2016**, *10*, 6400.

[15] H. Cui, Z. Zhang, M. Shi, Y. Xu, Y. Wu, *Anal. Chem.* **2005**, *77*, 6402.

[16] C. Lim, Y. Lee, J. Na, J. M. Oh, S. Her, K. Kim, K. Choi, S. Kim, I. C. Kwon, *Adv. Funct. Mater.* **2010**, *20*, 2644.

[17] Y. Zhang, X. Yang, Z. Gao, *RSC Adv.* **2015**, *5*, 21675.

[18] X. Shan, L. Chai, J. Ma, Z. Qian, J. Chen, H. Feng, *Analyst*, **2014**, *139*, 2322.

[19] L. Zhang, D. Peng, R. Liang, J. Qiu, *Chem. Eur. J.* **2015**, *21*, 9343.

[20] C. Shen, L. Su, J. Zang, X. Li, Q. Lou, C. Shan, *Nanoscale Res. Lett.* **2017**, *12*, 447.

[21] A. Zheng, Z. Cong, J. Wang, J. Li , H. Yang, G. Chen, *Biosens. Bioelectron.* **2013**, *49*, 519.

[22] F. Honarasa, F. H. Kamshoori, S. Fathi, Z. Motamedifar, *Microchim. Acta* **2019**, *186*, 234.

[23] Y. Dong, J. Zhang, P. Jiang, G. Wang, X. Wu, H. Zhao, C. Zhang, *New J. Chem*. **2015**, *39*, 4141.

[24] H. Tran, T. Nguyen, N. Nguyen, B. Piro, C. Huynh, *Microchim. Acta* **2018**, *185*, 270.

[25] N. Nirala, S. Abraham, V. Kumar, A. Bansal, A. Srivastav, P. Saxena, *Sens. Actuators B* **2015,** *218*, 42.

[26] S. Chen, X. Hai, X. Chen, J. Wang, *Anal. Chem*. **2014**, *86*, 6689.

[27] L. Lin, X. Song, Y. Chen, M. Rong, T. Zhao, Y. Wang, Y. Jiang, X. Chen, *Anal. Chim. Acta* **2015**, *869*, 89.

[28] Y. Long, X. Wang, D. Shen, H. Zheng, *Talanta* **2016**, *159*, 122.

[29] W. Shi, Q. Wang, Y. Long, Z. Cheng, S. Chen, H. Zheng, Y. Huang, *Chem. Commun*. **2011**, *47*, 6695.
